# Vici syndrome: a review

**DOI:** 10.1186/s13023-016-0399-x

**Published:** 2016-02-29

**Authors:** Susan Byrne, Carlo Dionisi-Vici, Luke Smith, Mathias Gautel, Heinz Jungbluth

**Affiliations:** Department of Paediatric Neurology, Neuromuscular Service, Evelina’s Children Hospital, Guy’s & St. Thomas’ Hospital NHS Foundation Trust, London, UK; Division of Metabolism and Laboratory of Molecular Medicine, Bambino Gesu Children’s Hospital IRCCS, Rome, Italy; Randall Division of Cell and Molecular Biophysics, Muscle Signalling Section, King’s College, London, UK; Department of Clinical and Basic Neuroscience, IoPPN, King’s College, London, UK

## Abstract

Vici syndrome [OMIM242840] is a severe, recessively inherited congenital disorder characterized by the principal features of callosal agenesis, cataracts, oculocutaneous hypopigmentation, cardiomyopathy, and a combined immunodeficiency. Profound developmental delay, progressive failure to thrive and acquired microcephaly are almost universal, suggesting an evolving (neuro) degenerative component. In most patients there is additional variable multisystem involvement that may affect virtually any organ system, including lungs, thyroid, liver and kidneys. A skeletal myopathy is consistently associated, and characterized by marked fibre type disproportion, increase in internal nuclei, numerous vacuoles, abnormal mitochondria and glycogen storage. Life expectancy is markedly reduced.

Vici syndrome is due to recessive mutations in *EPG5* on chromosome 18q12.3, encoding ectopic P granules protein 5 (EPG5), a key autophagy regulator in higher organisms. Autophagy is a fundamental cellular degradative pathway conserved throughout evolution with important roles in the removal of defective proteins and organelles, defence against infections and adaptation to changing metabolic demands. Almost 40 *EPG* mutations have been identified to date, most of them truncating and private to individual families.

The differential diagnosis of Vici syndrome includes a number of syndromes with overlapping clinical features, neurological and metabolic disorders with shared CNS abnormalities (in particular callosal agenesis), and primary neuromuscular disorders with a similar muscle biopsy appearance. Vici syndrome is also the most typical example of a novel group of inherited neurometabolic conditions, *congenital disorders of autophagy*.

Management is currently largely supportive and symptomatic but better understanding of the underlying autophagy defect will hopefully inform the development of targeted therapies in future.

## Disease name

Vici syndrome; Dionisi-Vici-Sabetta-Gambarara syndrome; Immunodeficiency with cleft lip/palate, cataract, hypopigmentation and absent corpus callosum.

## Definition

Vici syndrome [OMIM242840, ORPHA1493] is a severe congenital multisystem disorder characterized by the principal features of agenesis of the corpus callosum, cataracts, oculocutaneous hypopigmentation, cardiomyopathy, a combined immunodeficiency and additional, more variable multisystem involvement. The condition is due to recessive mutations in the *EPG5* gene on chromosome 18q.

## Epidemiology

The incidence of Vici syndrome is unknown. Since the original description of the disorder by Dionisi-Vici and colleagues in 1988 [1], an exponentially increasing number of patients has been reported, with around 50 genetically confirmed cases published to date [1–14]. Vici syndrome is likely to be rare but probably underdiagnosed.

## Clinical description

**Fig. 1:**
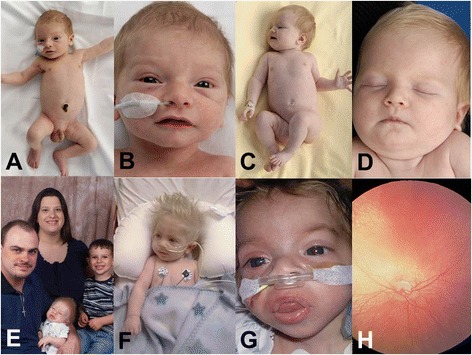
Clinical features of Vici syndrome. Note hypopigmentation in relation to ethnic (**a**–**d**, of Turkish origin) and familial (**e**–**f**) background. Coarsening of facial features with full lips and macroglossia resembling (lysosomal) storage disorders is noted in some older children (**g**). There is evidence of retinal hypopigmentation and optic atrophy on fundoscopy (**h**). From Cullup et al. *Nature Genetics* 2013; 45 (1):83–87, reproduced with permission

Vici syndrome is one of the most extensive inherited human multisystem disorders reported to date, presenting invariably in the first months of life. Apart from the 5 principal diagnostic findings–callosal agenesis, cataracts, cardiomyopathy, hypopigmentation and combined immunodeficiency-a wide range of variably present additional features has been reported, suggesting that virtually any organ system can be involved [4]. Three additional findings (profound developmental delay, acquired microcephaly and marked failure to thrive) have recently emerged that, although non-specific, are as consistently associated as the 5 main diagnostic features and highly supportive of the diagnosis [14]. The common occurrence of structural congenital abnormalities and acquired organ dysfunction (for example, congenital cardiac defects and cardiomyopathy later in life) is not infrequently observed in individual patients. Typical findings in Vici syndrome are outlined in detail below and summarized in Table 1. The characteristic features of Vici syndrome are illustrated in Fig. 1.

**Table 1:** Clinical features of Vici syndrome

	Feature	Frequency
*Principal diagnostic features*	*Absent corpus callosum*	++++
	Profound developmental delay	++++
	Failure to thrive	++++
	*Hypopigmentation*	++++
	*Immune problems*	++++
	Progressive microcephaly	+++
	*Cardiomyopathy*	+++
	*Cataracts*	+++
*Other features*	Presentation in neonatal period	+++
	Myopathy	+++
	Seizures	++
	Absent reflexes (probable neuropathy)	++
	Thymic aplasia	+
	Sensorineural deafness	+
	Optic atrophy	+
	Renal tubular acidosis	+
	Cleft lip/palate	+
	Coarse facial features	+
	Hepatomegaly	+

The 5 features initially considered to be diagnostic are indicated in italics. ++++ = present in almost all children, +++ = present in most children, ++ = present in more than half of children, + = present in some children

### CNS

Development in Vici syndrome is profoundly delayed: Affected children may acquire a social smile, some degree of head control, and the ability to roll over, however there have been no reports of children sitting independently, or acquiring speech. Where rolling has been attained, this skill may subsequently be lost. Almost two third of patients have seizures that are often difficult to control. Although head circumference is usually normal at birth, rapidly progressive microcephaly evolving within the first year of life suggests a neurodegenerative component superimposed on the principal neurodevelopmental defect.

In addition to agenesis of the corpus callosum, one of the five principal diagnostic features of Vici syndrome, other consistent radiological abnormalities include pontine hypoplasia, reduced opercularisation of the Sylvian fissures, delayed myelination and general reduction in white matter bulk [14]. Cortical malformations and cerebellar abnormalities have been observed but are much less common. In few patients, distinct circumscribed signal abnormalities (decrease in T2 with or without associated increase in T1 signal) have been noted within the thalami, similar to what has been described in patients with lysosomal storage disorders [15], also corresponding to some clinical overlap with these conditions.

### Muscle

An associated skeletal muscle myopathy, already suggested by the presence of often profound hypotonia and variable hyperCKaemia in early case reports, was documented in detail by McClelland and colleagues in 2010 [7] and subsequently confirmed in other reports [2, 12]. Clinically, individuals with Vici syndrome are often profoundly hypotonic and weak, probably reflecting a combination of the progressive nature of the myopathy and/or ongoing neurodegeneration. Histopathologically, the myopathy associated with Vici syndrome is characterized by marked variability in fibre size, increase in internal and centralized nuclei, type 1 fibre hypotrophy with normally sized type 2 fibres (occasionally fulfilling the criteria for fibre type disproportion), increased glycogen storage and variable vacuoles on light microscopy [2, 7, 14]. Additional changes on electron microscopy may include abnormalities of mitochondrial structure and arrangement [4, 14] and, less frequently, sarcomeric disorganization. On the histopathological level there is considerable overlap with the congenital myopathies, in particular Congenital Fibre Type Disproportion (CFTD) and Centronuclear Myopathy (CNM), primary vacuolar myopathies, glycogen storage disorders and mitochondrial myopathies.

### Nerves

Peripheral nerve involvement with almost complete absence of myelinated axons has been reported in only one case to date [14]; however, an associated neuropathy may have been overlooked in other patients because of the overwhelming nature of other multisystem features. The majority of children have absent deep tendon reflexes but those may be brisk in around a third.

### Skin

Marked oculocutaneous hypopigmentation [16] is one of the cardinal features of Vici syndrome and has been noted in almost all cases reported to date. Affected individuals are, however, not typically complete albinos and hypopigmentation is always relative to the familial and ethnic background (Fig. 1). Children with Vici syndrome have generally pale skin with light (often very blonde in those of Caucasian origin) hair, rather than discrete hypopigmented patches. An intermittent, extensive maculopapular rash almost resembling Stevens-Johnson syndrome has been reported in few children [14].

### Eyes

Bilateral cataracts are one of the “classical” diagnostic features of Vici syndrome, however, in a recent series of 50 patients those were only documented in three-quarters of affected individuals [14], probably reflecting evolution over time. Ocular features of Vici syndrome have been reviewed in detail by Filloux and colleagues [16] and include optic nerve hypoplasia, visual impairment, nystagmus and fundus hypopigmentation. Although individuals with Vici syndrome are usually only relatively hypopigmented, ocular features, in particular evidence of optic pathways misrouting on visually evoked potential (VEP) testing, and of a poorly defined and lesser depressed fovea on optical coherence tomography, are similar to those in individuals with typical albinism [16].

### Hearing

Sensorineural hearing loss was recognized in an isolated case in 2010 [7] and has been subsequently reported in other cases [6, 10] of Vici syndrome with or without confirmed *EPG5* mutations. Sensorineural hearing loss is a feature that may be easily overlooked in Vici syndrome due to profound developmental delay and overwhelming multisystem involvement, and should be actively investigated for.

### Heart

Cardiac involvement is present in around 90 % of patients with Vici syndrome and in around 80 % of cases a cardiomyopathy, one of the 5 main diagnostic features, has been documented. Minor congenital heart defects comprising persistent foramen ovale and atrial septal defects have been reported in around 10 % of patients. The associated cardiomyopathy usually develops early in life, although onset much later in childhood has been observed. Intermittent deterioration of cardiac function during intercurrent illness has also been noted (Patient 12.1 in [4]). Both hypertrophic and dilated forms of cardiomyopathy have been reported, always with left ventricular emphasis and occasionally in the same patient subsequently evolving over time. In two unrelated patients where post mortem examination was performed [8, 11], changes in the heart also showed left ventricular emphasis, with variable degrees of interstitial fibrosis and cardiomyocytes containing vacuoles and membrane-bound cytoplasmic inclusions, possibly glycogen. In keeping with the underlying autophagy defect, cardiomyocytes showed increased staining for autophagy markers LC3 and p62 on immunohistochemistry [8].

### Immune system

A combined immunodeficiency is one of the diagnostic hallmarks of Vici syndrome but is highly variable, mainly depending on age and ranging from near normal to severely compromised immunity (for review, [6]). The associated immune defect manifests as recurrent, commonly respiratory, infections from early in life, also including mucocutaneous candidiasis, sepsis and, less frequently, urinary tract infections, gastroenteritis, bacterial conjunctivitis, and perineal abscesses. Due to the severely reduced life expectancy, immune function has been assessed formally only in a few patients [6]. Abnormal findings reported to date include lymphopenia with variable T cell subset defects, neutropenia, leucopenia, hypogammaglobulinaemia, lack of response to recall antigens and a defect of memory B cells with lack of specific antibody response to certain immunizations such as those with *tetanus* and pneumococcal vaccine. Overall, these findings suggest prominent impairment of the humoral immune response with a milder defect of the T cell compartment, although further prospective studies will be required to delineate the immunological phenotype further. Immunological features of Vici syndrome, recommended immunological investigations and potential treatment approaches have been outlined in detail by Finocchi and colleagues [6].

### Thymus

Complete thymus aplasia or hypoplasia has been reported in around one fifth of patients [4, 14]. T-cell dysfunction is part of the combined immunodeficiency observed in Vici syndrome although usually less prominent than B-cell dysfunction [6].

### Lungs

Pulmonary hypoplasia has been reported in one patient with Vici syndrome [2]. Pulmonary involvement is common throughout life, due to recurrent respiratory infections secondary to the associated combined immunodeficiency.

### Thyroid

Thyroid agenesis and thyroid dysfunction have both been reported in rare patients with Vici syndrome [4, 14].

### Liver

Hepatomegaly with or without associated liver dysfunction has been reported in around 10 % of patients with Vici syndrome [4, 14] and is probably a reflection of increased glycogen storage, also reported on post mortem in few cases.

### Kidneys

Renal involvement comprising hydronephrosis, renal dysfunction and/or signs of renal tubular acidosis with associated electrolyte imbalances, in particular marked hypokalaemia, have been reported in around 15 % of cases [4, 9, 14].

### Blood

Some patients with Vici syndrome have been noted to develop profound anaemia [4, 14]; it is currently uncertain if this is a secondary feature (for example related to recurrent severe infections) or, alternatively, reflects additional primary involvement of red cell lines.

### Other features

Mildly dysmorphic, coarse facial features with full lips and macroglossia resembling those seen in (lysosomal) storage disorders have been noted in some patients with Vici syndrome [4, 14] (Fig. 1). Cleft lip and palate were a feature in Dionisi-Vici’s original siblings [1] but have subsequently been seen only in few families. Other minor dysmorphic features such as 2^nd^ and 3^rd^ toe syndactyly were a feature in two families reported [4, 14]. A long philtrum has been described in one family [17]. Marked failure to thrive evolving over time has been recently recognized as an almost universal feature [14]. One recent case report also suggests severe sleep abnormalities that may have to be considered in Vici syndrome [18].

### Aetiology

Vici syndrome is due to recessive mutations in *EPG5* on chromosome 18q12.3, organized in 44 exons and encoding ectopic P granules protein 5 (EPG5), a protein of 2579 amino acids. *EPG5* (originally known as *KIAA1632*) was initially identified amongst a group of genes found to be mutated in breast cancer tissue [19] before its implication in Vici syndrome in 2013 [4].

To date, around 40 *EPG5* mutations have been identified in families with Vici syndrome, distributed throughout the entire *EPG5* coding sequence without clear genotype-phenotype correlations [13, 14]. Most *EPG5* mutations associated with Vici syndrome are truncating with only few missense mutations on record. The large majority of *EPG5* mutations are private to individual families, with only 3 recurrent mutations identified to date, p. Met2242CysfsX5 in an Italian and a Maltese family, p. Arg417X identified in the homozygous state in a patient from the Middle East and in the heterozygous state in a Caucasian child from the United States, and p. Gln336Arg identified in the homozygous (*n* = 3) and in the heterozygous (*n* = 1) state in four unrelated patients with definite or possible Ashkenazi ancestry [14]. Failure to identify an (or identification of one but not the allelic) *EPG5* mutation in a small number of cases with highly suggestive diagnostic features indicate the possibility of large copy number variations not detectable on Sanger sequencing, or an altogether different genetic background.

**Fig. 2:**
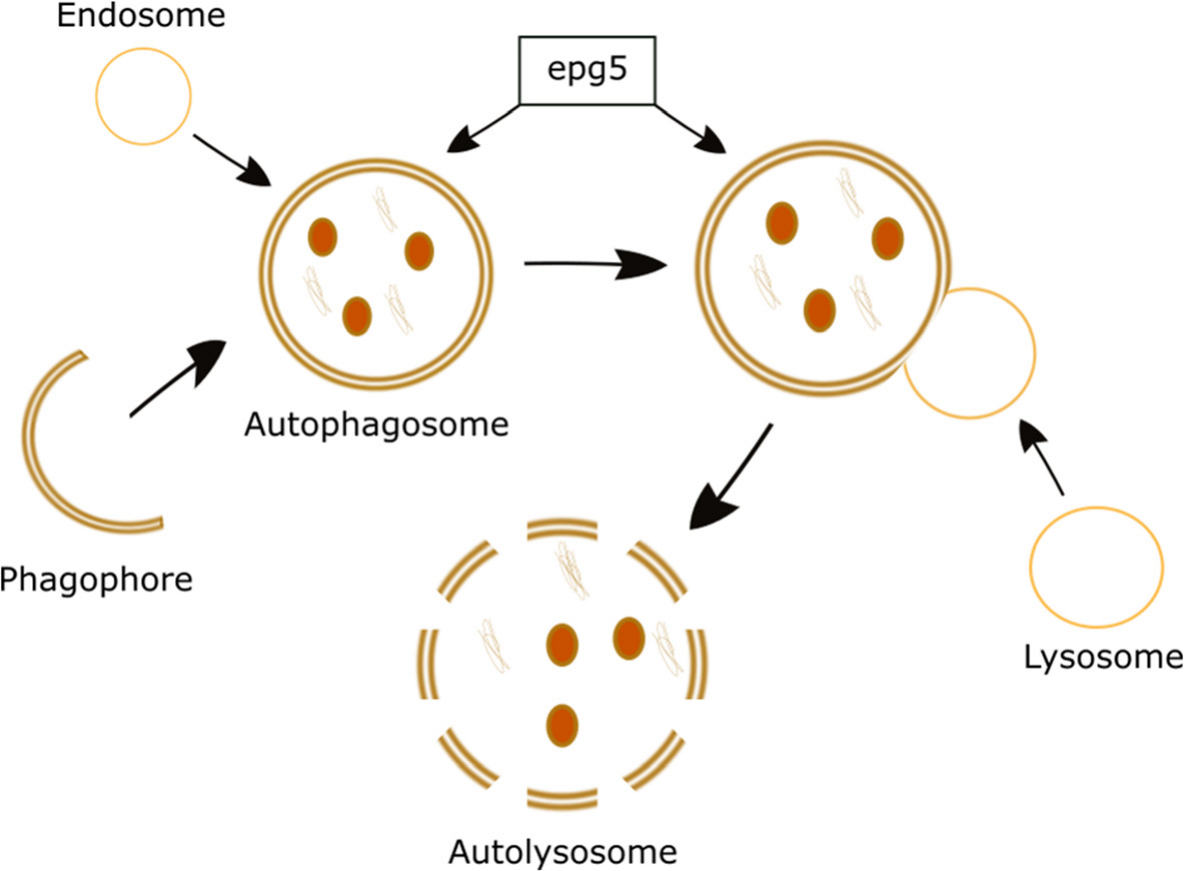
Schematic representation of the autophagy pathway. The autophagy pathway involves several tightly regulated steps, evolving from the initial formation of phagophores to autophagosomes, whose fusion with lysosomes results in the final structures of degradation, autolysosomes. The EPG5 protein plays a role in fusion events between autophagosomes, lysosomes, and, possibly, endosomes

The EPG5 protein has a key role as a regulator of autophagy in multicellular organisms, initially characterized in C. elegans [20] and subsequently confirmed in *EPG5*-mutated humans with Vici syndrome [4]. Autophagy is a fundamental cellular degradative pathway conserved throughout evolution with important roles in the removal of defective proteins and organelles, defence against infections and adaptation to changing metabolic demands (for review [[21–23]]). The autophagy pathway involves several tightly regulated steps, evolving from the initial formation of isolation membranes (or phagophores) to autophagosomes, whose fusion with lysosomes results in the final structures of degradation, autolysosomes (Fig. 2). The ultimate aim of the autophagy pathway is the effective delivery of an intracellular structure targeted for removal to the lysosome, and its ultimate intralysosomal degradation. Studies in *EPG5*-mutated fibroblasts from humans with Vici syndrome suggest that EPG5 deficiency results in failure of autophagosome-lysosome fusion [4] and, ultimately, impaired cargo delivery to the lysosome. It is currently uncertain if impaired autophagy is the only consequence of EPG5 deficiency, or only the most important expression of a more generalized vesicular trafficking defect in Vici syndrome. Moreover, it remains unresolved if all manifestations of EPG5 deficiency are a direct consequence of the primary autophagy defect, or of the secondary effects of defective autophagy such as reduced mitochondrial quality control and/or accumulation of defective proteins.

Autophagy is physiologically enhanced in neurons and muscle, probably explaining the prominent CNS and neuromuscular involvement in patients with Vici syndrome and other conditions with primary autophagy defects. The phenotype of epg5-/-KO mice recapitulates the autophagy defect and the skeletal muscle myopathy seen in humans with Vici syndrome [24], and in addition exhibits clinical and pathological neurodegenerative features, in particular progressive motor deficit, muscle atrophy and damage of cortical 5 layer and spinal motor neurones, resembling human amyotrophic lateral sclerosis (ALS). A recently generated conditional drosophila knockout also shows a marked autophagy defect and evidence of progressive neurodegeneration in retinal photoneurons [14]. Taken together, these findings indicate Vici syndrome as a paradigm of a disorder linking neurodevelopment and neurodegeneration in the same pathway. Following the genetic resolution of Vici syndrome in 2013, a number of disorders associated with defects in primary autophagy regulators have now been identified–for example, Static Encephalopathy in childhood with NeuroDegeneration in Adulthood (SENDA) due to X-linked recessive mutations in *WDR45*, and early-onset syndromic ataxia due to recessive mutations in *SNX14*, suggesting *congenital disorders of autophagy* as a novel group of neurometabolic disorders with recognizable features, mechanistically linked in the same pathway (reviewed in, [25]).

Apart from the heart, the role of normally functioning autophagy in other organ systems involved in Vici syndrome has been much less explored but poses interesting questions for future research, regarding the normal biology of organ development but also organ-specific disease.

### Diagnosis

The diagnosis of Vici syndrome is based on the presence of suggestive clinical features and confirmation of recessive *EPG5* mutations on diagnostic genetic testing. Based on binary logistic regression analysis, the presence of the eight key features as outlined above (absent corpus callosum, cataracts, hypopigmentation, cardiomyopathy, immune dysfunction, profound developmental delay, progressive microcephaly, failure to thrive) has a specificity of 97 %, and a sensitivity of 89 % for a positive *EPG5* genetic test [14]. *EPG5* testing is now offered as a diagnostic service [4]. Although the vast majority of *EPG5* mutations is unequivocally pathogenic, rarely *EPG5* variants of uncertain significance may require functional autophagy studies in fibroblast cultures that are currently only available on a research basis. In addition, introduction of complementary diagnostic genetic strategies (including high resolution CHG arrays, targeted MLPA testing, RNA studies) to investigate the possibility of copy number variations within the large *EPG5* gene are indicated in patients with suggestive diagnostic features where only one or no clearly pathogenic *EPG5* variants have been identified on Sanger sequencing.

Other useful diagnostic investigations to document the extent of multisystem involvement (summarized in Table 2) include an MRI of the brain (in particular to document the callosal agenesis, one of the key diagnostic features), EEG, ophthalmology assesment including slit lamp examination and VEPs, chest x-ray, cardiac assessment including cardiac ultrasound, an abdominal ultrasound to document the extent of organ involvement, laboratory investigations assessing immune, thyroid, liver and renal function (see also paragraph on management). A muscle biopsy is not strictly needed to establish the diagnosis, however, in cases where this was performed before Vici syndrome was suspected, a certain combination of consistent histopathological features as outlined above may be supportive of *EPG5* involvement.

**Table 2:** Recommended investigations for the diagnosis and surveillance of patients with Vici syndrome

Investigation	Presentation/Diagnosis [*expected key findings*]	Surveillance
*EPG5 testing*	Baseline investigation [*homozygous/ compound heterozygous mutation*]	Not required
*MRI brain*	Baseline investigation [*Congenital absence of corpus callosum, along with other described features*]^a^	Not routinely required
*Ophthalmology assessment*	Baseline investigation [*Cataracts, ocular albinism*]^b^	Required surveillance for cataracts
*Cardiac ultrasound*	Baseline investigation [*Structural defects and/or cardiomyopathy*]^a^	Required surveillance for progressive cardiomyopathy
*Chest x-ray*	Baseline investigation [*Thymus aplasia/hypoplasia*]	If clinically indicated
*Immune function tests*	Baseline investigation^c^	Required surveillance for progressive immunedeficiency
*Renal function tests*	Baseline investigation	If clinically indicated
*Thyroid function tests*	Baseline investigation	If clinically indicated
*Liver function tests*	Baseline investigation	If clinically indicated
*Amino acids assessment*	Baseline investigation	If clinically indicated
*Feeding study*	Often clinically indicated [*most children require percutaneous feeding*]	If clinically indicated
*EEG*	If clinically indicated	If clinically indicated
*Sleep study*	If clinically indicated	If clinically indicated
*Muscle biopsy*	No longer indicated if genetic diagnosis has been established^a^	No longer indicated if genetic diagnosis has been established

For more detail of recommended investigations and/or expected findings see ^a^[14] ^b^[16] ^c^[6]

### Differential diagnosis

Although in the presence of all principal features the clinical diagnosis of Vici syndrome should be straightforward and prompt *EPG5* testing, it is important to bear in mind that some of these features (in particular cataracts, cardiomyopathy and immunodeficiency) may only evolve over time and are not necessarily present from birth. The differential diagnosis of Vici syndrome includes a number of syndromes with overlapping clinical features, neurological and metabolic disorders with similar CNS abnormalities (in particular callosal agenesis) and primary neuromuscular disorders with a similar muscle biopsy appearance.

Amongst the syndromic conditions that may mimic Vici syndrome (Table 3), Marinesco-Sjoegren syndrome (MSS) and related disorders share cataracts and a skeletal muscle myopathy with or without sensorineural deafness; however, failure to thrive and acquired microcephaly are uncommon and the degree of developmental delay is also usually less severe [26]. Hypopigmentation and immune defects are the typical features of Chédiak-Higashi (CHS) syndrome and related primary immunodeficiency syndromes. Amongst the latter group, Griscelli syndrome (GS) most closely resembles Vici syndrome, and is further subdivided in 3 clinically and genetically distinct groups (for review, [27]), of which only GS type 2 due to recessive mutations in *RAB27A* features prominent immunological involvement and hemophagocytic lymphohistiocytosis (HLH), whereas GS type 1 due to recessive *MYO5A*, the allelic Elejalde syndrome (ES) and GS type 3 due to recessive *MLPH* mutations only feature pigmentary abnormalities with or without primary neurological features, respectively, but not typically immunodeficiency. Interestingly, at least in a subset of patients, MSS, CHS, GS and ES are also neurodevelopmental disoders that, in common with Vici syndrome, may develop clinical features of early-onset neurodegeneration [28–32].

**Table 3:** Syndromes showing phenotypical overlap with Vici syndrome (selection)

Condition	Gene	Clinical feature						
		CNS	Cataract	Cardiomyopathy	Myopathy	Neuropathy	Immunodeficiency	Hypopigmentation
Vici syndrome	*EPG5*	+	+	+	+	+	+	+
MSS	*SIL1*	+	+	−	+	+^a^	−	−
CCFDN	*CTDP1*	+	+	−	+	+	−	−
Nathalie syndrome	?	+	+	+	+	−	−	−
Griscelli syndrome 1	*MYO5A*	+	−	−	?	−	−	+
Griscelli syndrome 2	*RAB27A*	+	−	−	?	−	+	+
Griscelli syndrome 3	*MLPH*	−	−	−	?	−	−	+
Elejalde syndrome	*RAB27A*	+	−	−	?	−	−	+
CHS	*LYST*	+	−	−	+	(+)	+	+
HPS 2	*AP3B1*	+	−	−	?	+	−	+
Cohen syndrome	*VPS13B*	+	−	(+)	−	−	+	−
Danon disease	*LAMP2*	+	−	+	+	+	−	−
MEDNIK	*AP1S1*	+	(+)	−	−	+	−	−
CEDNIK	*SNAP29*	+	+	−	−	+	−	−

*MSS* marinesco-sjoegren syndrome, *CCFDN* congenital cataracts, facial dysmorphism and neuropathy syndrome, *CHS* chediak-higashi syndrome, *HPS*2 hermanksy-pudlak syndrome type 2, *MEDNIK* mental retardation, enteropathy, deafness, peripheral neuropathy, ichthyosis and keratoderma syndrome. + = feature present; - = feature absent; ? = not specifically investigated; (+) = feature controversial or not sufficiently documented; ^a^ = neuronopathy

On the neuroradiological level, the differential diagnosis of callosal agenesis is wide and in relation to Vici syndrome has been summarized by McClelland et al. [7]. Thalamic changes in some patients with Vici syndrome may resemble those seen in patients with primary (lysosomal) storage disorders [15], a group of conditions also featuring some clinical overlap.

On the histopathological level, muscle biopsy findings in Vici syndrome may mimic a number of primary neuromuscular disorders, in particular vacuolar myopathies [33] and the centronuclear myopathies [34], conditions that, interestingly, have been linked with primary and secondary defects of the autophagy pathway [35]. The defects implicated in Danon disease [36] and X-linked myopathy with excessive autophagy (MEAX) [37], in particular impaired autolysosomal fusion and defective intralysosomal digestion, concern the same part of the autophagy pathway also affected in Vici syndrome. Considering common features of increased glycogen storage and abnormal mitochondria, Vici syndrome (or indeed other disorders with primary autophagy defects) also ought to be considered in patients with suspected but genetically unresolved glycogen or mitochondrial disorder.

### Management

There is currently no cure for Vici syndrome and management is essentially supportive, aimed at alleviating the effects of extensive multisystem involvement.

As some of the associated features may only evolve over time, in addition to their usefulness at the point of diagnosis, investigations that ought to be repeated at an interval include EEG, ophthalmology assesment including slit lamp examination, CXR, cardiac assessment including cardiac ultrasound, and laboratory investigations assessing immune, thyroid, liver and renal function (see also paragraph on diagnosis). Investigations recommended in patients with suspected or established Vici syndrome are summarized in Table 2.

Management of the associated *immunodeficiency* poses a particular challenge and may require regular intravenous immunoglobulin infusions and antimicrobial prophylaxis. It is also important to bear in mind that patients with Vici syndrome may fail to respond to certain immunizations such as those with tetanus or pneumococcal vaccines. An detailed overview of recommended immunological investigations and possible management approaches is provided by Finocchi et al. [6].

More than half of patients with Vici syndrome have *seizures* that ought to be managed with appropriate anticonvulsant therapy. Considering the profound autophagy abnormalities observed in patients with Vici syndrome, responses to anticonvulsants (or, indeed, other drugs) with potentially autophagy-modulating properties such as carbamazepine should perhaps be monitored closely following initiation of treatment.

If *cataracts* are present surgical removal may improve visual outcome but the indication for cataract surgery will have to be decided on an individual basis, based on overall severity and expected prognosis.

If a cardiomyopathy is identified on regular cardiac assessments, this may benefit from proactive medical management; a deterioration of cardiac function during intercurrent illness has to be expected. Both *central and obstructive* apnoea may require polysomnographic monitoring, and non-invasive ventilatory support as indicated.

*Hypothyroidism* may require thyroid hormone replacement. Renal dysfunction and *electrolyte imbalances*, in particular profound hypokalaemia, will have to be anticipated and managed actively. Profound *anaemia* may require blood transfusion in some patients.

### Counselling

Vici syndrome is inherited in an autosomal-recessive fashion. Genetic counselling should be offered to all families in whom a diagnosis of Vici syndrome has been established. Mutational analysis of the *EPG5* gene is now available on a diagnostic basis [4], and *EPG5* testing, the gold standard of antenatal diagnosis, can be offered to families where causative *EPG5* mutations have been identified. It is important to bear in mind that foetal ultrasound applied for the detection of callosal agenesis may yield false positive and false negative results, therefore when genetic testing is not readily available foetal MRI ought to be the preferred form of imaging.

### Prognosis

Vici syndrome is a relentlessly progressive condition and survival beyond the first decade has not been reported. A large series recently demonstrated that death occurred at a median age of 42 months (range 1 to 102 months). Patients with homozygous mutations died sooner than patients with heterozygous mutations (median age nine months compared to 48 months) [14]. The degree of cardiac involvement and/or the extent of the associated immunodeficiency are the most important prognostic indicators.

### Unresolved questions

Vici syndrome is the most extensive human multisystem disorder attributed to a primary autophagy defect reported to date. Although rare, the condition illustrates the impact of defective autophagy not only on neurodevelopment and neurodegeneration but also on a wide range of other organ systems where the role of normally functioning autophagy is currently only partially understood or not even considered yet. There are a number of unresolved questions of direct relevance to families affected by Vici syndrome but also for the wider field of autophagy research:

It is currently uncertain if Vici syndrome is genetically homogeneous, with the failure to identify two allelic mutations in some patients due to *EPG5* copy number variations not detectable on Sanger sequencing, or if there is genuine genetic heterogeneity with novel genetic backgrounds yet to be discovered in individuals with suggestive features but no *EPG5* mutations identified. Little is known about the physiological cellular interactions of the EPG5 protein, and it remains unclear if impaired autophagy is the only consequence of EPG5 deficiency, or just the most dramatic expression of a more generalized vesicular trafficking defect in patients with Vici syndrome. The autophagy pathway is amenable to pharmacological manipulation, and delineating the precise defect in Vici syndrome will be important for the development of rational therapies in future. The marked phenotypical overlap between Vici and clinically related syndromes such as MSS or CHS is currently unexplained but suggests potential interaction of the defective proteins in related cellular pathways, resulting in similar phenotypes.

Identification of new genotypes, further characterization of the precise biological role of EPG5 and the relation between Vici and similar syndromes will further elucidate the role of defective autophagy in inherited multisystem disorders, and hopefully result in the development of effective therapies for Vici syndrome and related conditions in future.

### Consent

Written informed consent was obtained from the patient (s) for publication of this manuscript and accompanying images. A copy of the written consent is available for review by the Editor-in-Chief of this journal.
